# SFMBT1 facilitates colon cancer cell metastasis and drug resistance combined with HMG20A

**DOI:** 10.1038/s41420-022-01057-7

**Published:** 2022-05-16

**Authors:** Ruijun Pan, Dingye Yu, Jiajia Hu, Xiao Yang, Chenxing Wang, Luyang Zhang, Pei Xue, Jing Sun, Xiaoping Zhang, Wei Cai

**Affiliations:** 1 Department of General Surgery, Ruijin Hospital, Shanghai Jiao Tong University School of Medicine, Shanghai, China; 2 Shanghai Minimally Invasive Surgery Center, Shanghai, China; 3 Department of Nuclear Medicine, Ruijin Hospital, Shanghai Jiao Tong University School of Medicine, Shanghai, China; 4 Department of Interventional & Vascular Surgery, Tenth People’s Hospital of Tongji University, Shanghai, 200072 China; 5 Institute of Interventional & Vascular Surgery, Tongji University, Shanghai, 200072 China

**Keywords:** Metastasis, Cancer therapy

## Abstract

In colorectal cancer (CRC), the development of reagents that increase sensitivity to chemotherapeutic agents could prevent drug resistance and improve patient survival. Scm-like with four malignant brain tumor domains 1 (SFMBT1) is up-regulated in CRC tumor tissues and cells and may be associated with drug resistance. We detected the expression of SFMBT1 in CRC tissue microarrays by immunohistochemistry. The role of SFMBT1 in the migration, proliferation and invasion of CRC or resistance to 5-fluorouracil (5-FU) was determined using scratch assay, colony formation and Transwell assay. Fluorescence co-localization and immunoprecipitation were used to analyze the correlation between SFMBT1 and high mobility group domain-containing protein 20 A (HMG20A). Xenograft experiments were conducted to investigate the role of SFMBT1 and HMG20A in tumor growth and metastasis in vivo. We found that SFMBT1 is up-regulated in CRC and its expression is further amplified in 5-FU resistance. SFMBT1 drives 5-FU resistance and CRC proliferation, migration and invasion. Correlation analysis shows that SFMBT1 and HMG20A are positively correlated. Mechanistically, fluorescence co-localization and immunoprecipitation assay indicate an interaction between SFMBT1 and HMG20A. Depletion of SFMBT1 down-regulates HMG20A downstream. These results were verified by murine xenograft and lung metastasis models. Our results indicate that the SFMBT1/HMG20A axis could be targeted to increase the resistance of CRC cells to 5-FU.

## Introduction

Although patient outcomes have improved with the emergence of targeted drug therapies, colorectal cancer (CRC) is still one of the leading causes of fatalities associated with cancer worldwide [1–3]. The prognosis of patients with unresectable cancer combined with metastasis and drug resistance is particularly dismal [4, 5]. The uracil analog 5-fluorouracil (5-FU) is the chemotherapeutic drug predominantly used in the frontline treatment of CRC [6, 7]. However, 5-FU can have debilitating neurotoxic side effects in patients, therefore, it is necessary to maintain an effective dose at low concentrations [6, 8]. However, it is difficult to maintain an effective dose of 5-FU because the modification and altered expression of several genes, including MLH1, Bcl-2 and TR1 can lead to 5-FU resistance [9].

In particular, the altered expression of forkhead box O (FOXO) proteins is related to the resistance to antitumor agents, including 5-FU [10, 11]. The differential expression of FOXO1 is associated with resistance to 5-FU [11–13]. FOXO1 is a tumor repressor that is believed to be down-regulated in cancer through negative regulation by the enhancer of zeste homolog 2 (EZH2) [14, 15]. EZH2 promotes resistance to several drugs including 5-FU in CRC through the indirect activation of NF-κB signaling [16, 17]. Although some progress has been made in counteracting resistance to 5-FU, the response rates remain low [18]. Therefore, a better understanding of the mechanisms associated with drug resistance in CRC will provide the potential to develop appropriate therapeutic strategies.

Genome-wide analysis of genes that are differentially expressed in drug-resistant cells has advanced knowledge in this region substantially [19–21]. By performing association analysis with single nucleotide polymorphisms, Law et al. discovered 31 risk loci associated with CRC [19]. Scm-like with four malignant brain tumor domains 1 (SFMBT1), a polycomb protein that was found to be associated with diseases of the colon. SFMBT1 is a transcriptional repressor that associates with chromatin and interacts with the N-terminal tail of histone H3 [22]. SFMBT1 forms a complex with Lys-specific demethylase 1 (LSD1) and they are believed to act together in combination with RNA polymerase II in the transcriptional regulation of histone genes [23].

The high mobility group domain-containing protein 20 A (HMG20A) is also known to interact with LSD1 [24]. Cells depleted in HMG20A have increased lysine 4 methylation in histone H3 and are less invasive. The Cancer Genome Atlas (TCGA) indicates that SFMBT1 and HMG20A can interact. Therefore, in this study, we investigate the interactions between SFMBT1 and HMG20A and the impact of 5-FU in CRC cell lines and a mouse xenograft model of CRC tumorigenesis.

## Results

### SFMBT1 is up-regulated in CRC tumor tissues and cells

**Fig. 1:**
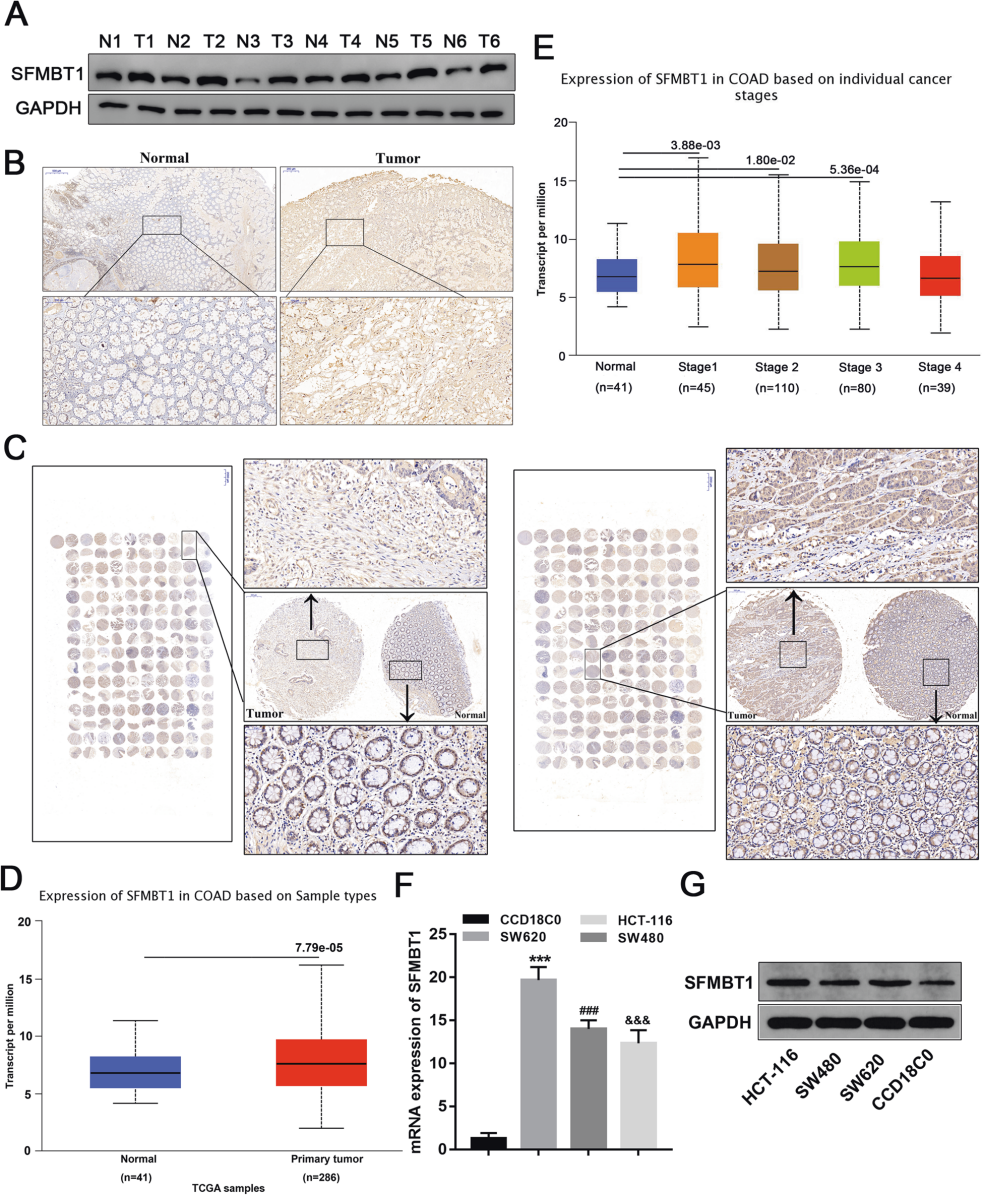
Distribution of SFMBT1 in colorectal cancer (CRC) tumor tissues and cells. **A** Western blot analysis of SFMBT1 protein levels in normal and tumor tissues. (*n* = 6) (**B**) Expression of SFMBT1 in normal and CRC tumor tissues were examined by immunohistochemistry. **C** Expression of SFMBT1 in CRC tissue microarrays. **D**, **E** TCGA data shows the expression of SFMBT1 in tumor tissues and normal tissues or in different cancer stages. **F**, **G** qRT-PCR (**F**) and western blot analysis (**G**) detect SFMBT1 mRNA and protein in human colorectal epithelial cells CCD18C0 and CRC cell lines HCT-116, SW620 and SW480.

To investigate the role of SFMBT1 in colon cancer, we first examined its expression in human colon cancer tissues. Compared with adjacent normal tissues, colon cancer tissues exhibited significantly higher SFMBT1 levels (Fig. 1A, and full length uncropped original western blots in Supplemental Material). Expression of SFMBT1 was also examined in CRC by immunohistochemistry (IHC) in tumor tissues (Fig. 1B) and tissue microarrays (Fig. 1C). The glandular regions in the CRC tumor tissues are poorly differentiated compared to normal tissue with clusters of tumor cells overexpressing SFMBT1. The disorganization of tissue can also be observed in CRC tissue microarrays. TCGA shows the expression of SFMBT1 in tumor tissues and adjacent normal tissues or in different stages of cancer (Fig. 1D, E). After discovering that SFMBT1 was expressed at higher levels in tumor tissues, we determined its expression in human colorectal epithelial cells and CRC cell lines. Western blot analysis confirmed that the expression levels of SFMBT1 in CRC cells were higher than in normal epithelial colorectal cells (Fig. 1F, G, and full length uncropped original western blots in Supplementary Material).

### SFMBT1 drives 5-FU resistance and CRC proliferation, migration and invasion

**Fig. 2:**
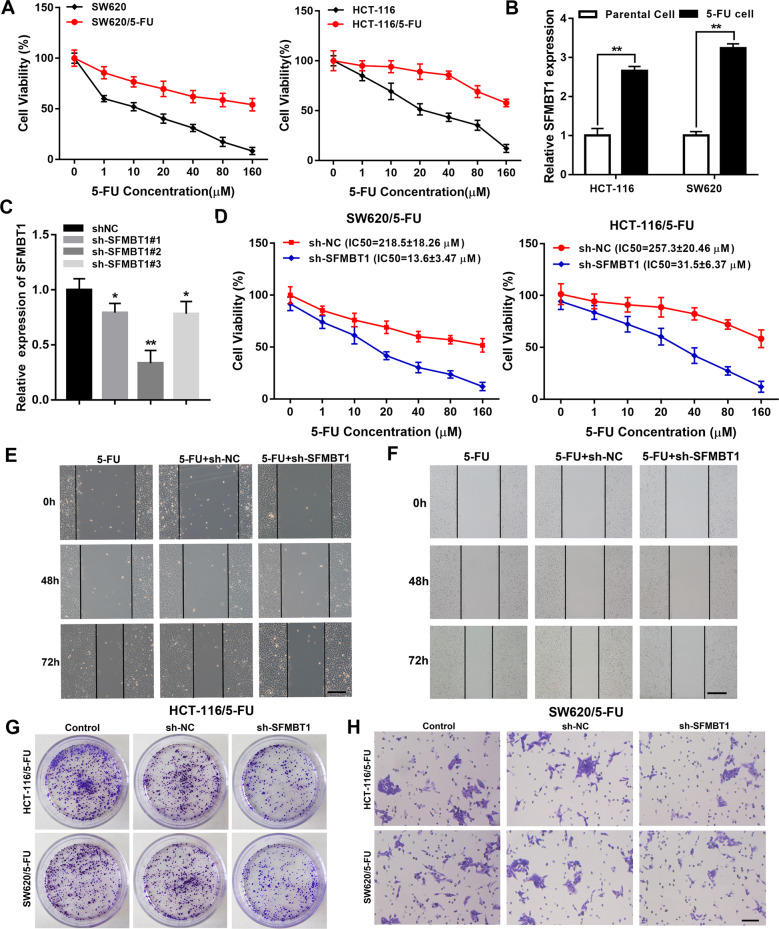
SFMBT1 drives 5-fluorouracil (5-FU) resistance and CRC proliferation, migration and invasion. **A** Cell viability with indicated concentrations of 5-FU was evaluated by MTT assay in parental and 5-FU resistance HCT-116 and SW620 cells. **B** SFMBT1 mRNA level in parental cells and 5-FU resistance cells. **C** Expression levels to determine the efficiency of SFMBT1 knockdown by qRT-PCR. *P < 0.05, **P < 0.01^.^ **D** Cell viability with indicated concentrations in HCT116/5-FU and SW620/5-FU after sh-NC or sh-SFMBT1 treatment by MTT assay. **E**, **F** Wound scratch assay to measure migration in HCT-116/5-FU (**E**) and SW620/5-FU (**F**) cells transfected with sh-NC or sh-SFMBT1. **G** Colony formation assay to measure proliferation in HCT-116/5-FU and SW620/5-FU cells transfected with sh-NC or sh-SFMBT1. **H** Transwell assay to measure invasion in HCT-116/5-FU and SW620/5-FU cells transfected with sh-NC or sh-SFMBT1.

We selected two 5-FU sensitive CRC cell lines (HCT116 and SW620 cells). By continuously increasing the concentration of 5-FU and calculated cell viability with indicated concentrations of 5-FU by MTT assay in parental and 5-FU resistance HCT-116 and SW620 cells (Fig. 2A), we obtained 5-FU-resistant CRC cells and named HCT-116/5-FU and SW620/5-FU. The qRT-PCR results showed that the levels of *SFMBT1* increased in HCT-116 and SW620 5-FU resistant cells (Fig. 2B). We subsequently transfected HCT-116 and SW620 cells with plasmids expressing sh-SFMBT1. The knockdown efficiency of *SFMBT1* was confirmed with qRT-PCR analysis (Fig. 2C). MTT assay indicated that the IC50 values in HCT-116/5-FU and SW620/5-FU cells after transfected with sh-NC or sh-SFMBT1, the results demonstrated that silencing of SFMBT1 significantly affected 5-FU resistant HCT-116 and SW620 cells (Fig. 2D). SFMBT1 knockdown in 5-FU resistant HCT-116 and SW620 cells inhibited proliferation, migration and invasion (Fig. 2E–H, Additional Fig. 1A–C).

### SFMBT1 knockdown in mouse xenograft model reduces drug resistance and metastasis

**Fig. 3:**
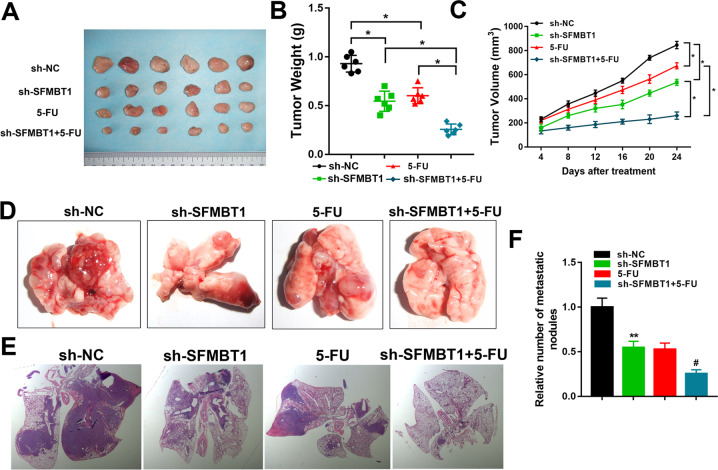
SFMBT1 knockdown in mouse xenograft model reduces drug resistance and metastasis. **A** Xenografts derived from nude mice subcutaneously injected with the indicated cells (*n* = 6). **B** Tumor weight of nude mice recorded every 4 days after the initial treatment. **C** Tumor volume after different treatments. **D**, **E** Representative images of lungs (**D**) from each group with H&E staining (**E**) of metastatic tissue sections. **F** The relative number of metastatic nodules were quantified. *P < 0.05.

We constructed a mouse xenograft model using HCT-116/5-FU cells with the expression of SFMBT1 silenced. Mice were treated with 5-FU and the tumor volume was observed for 24 days. The silencing of SFMBT1 significantly reduced tumor growth (Fig. 3A–C). In mice treated with 5-FU and implanted with SFMBT1 silenced cells, lung metastatic nodules (Fig. 3D, E) and number (Fig. 3F) were reduced. Consistent with in vitro experimental results, SFMBT1 promotes cancer tumorigenesis and 5-FU resistance of colon cancer in vivo.

### HMG20A is associated with SFMBT1 and its expression is increased in colorectal cancer tissues

**Fig. 4:**
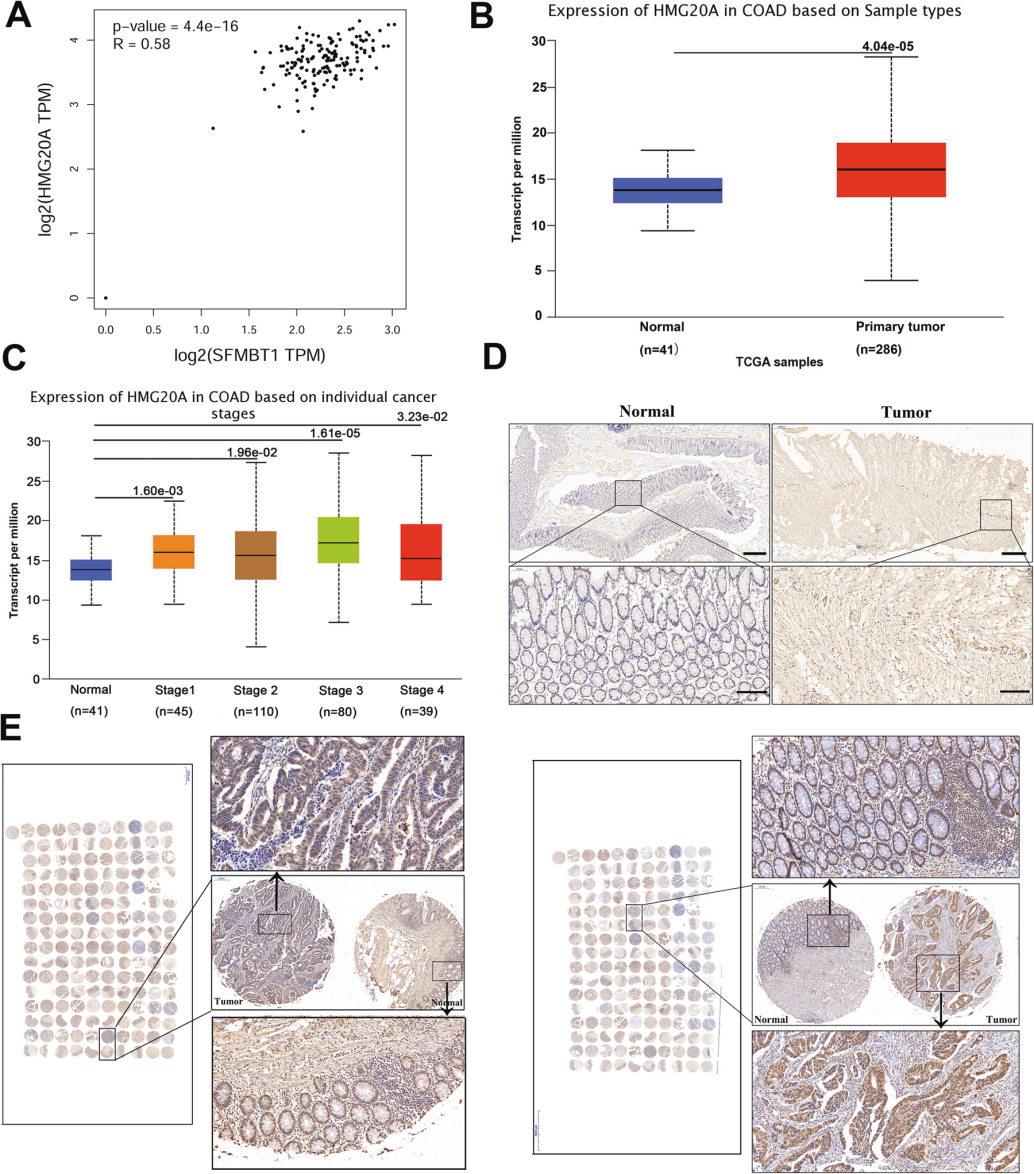
HMG20A is increased in colorectal cancer (CRC) tissue. **A** Correlation between SFMBT1 and HMG20A using GEPIA data. **B** TCGA data shows the expression of HMG20A in primary tumor tissues and normal tissues. **C** TCGA data shows the expression of HMG20A in tumor tissues and normal tissues in different cancer stages. **D** Expression of HMG20A in normal and CRC tumor tissues were examined by immunohistochemistry. **E** Expression of HMG20A in CRC tissue microarrays.

The clinicopathological characteristics associated with SFMBT1 and HMG20A in patients can be found in Table 1. Of note, SFMBT1 is positively correlated with HMG20A in the Gene Expression Profiling Interactive Analysis (GEPIA) database (Fig. 4A), suggesting a regulatory relationship. In TCGA, HMG20A is higher in tumor tissues than in adjacent normal tissues (Fig. 4B). The expression of HMG20A in normal tissues and tumor tissues of different cancer stages in TCGA (Fig. 4C). Expression of HMG20A was examined in CRC by IHC in tumor tissues (Fig. 4D) and tissue microarrays (Fig. 4E). The glandular regions in the CRC tumor tissues are poorly differentiated compared to normal tissues under the higher expression of HMG20A.

**Table 1:** SFMBT1 and HMG20A protein level and clinic parameters in 65 patients with colon cancer.

Protein name		SFMBT1		HMG20A	
Characteristics	All cases	Low	High	Low	High
Participants	65	28	37	30	35
Age (years)					
<60	22	10	12	8	14
≥60	43	18	25	16	27
Gender					
Male	35	16	19	15	20
Female	30	15	15	12	18
clinical stage					
1–2	42	20	22	15	27
3–4	23	8	15	11	12
Tumor size (mm3)					
<30	26	12	14	12	14
≥30	39	20	19	21	18
Lymphnode metastasis					
Negative	43	21	22	17	26
Positive	22	6	16	10	12

### HMG20A mediates 5-FU resistance and CRC proliferation, migration, and invasion

**Fig. 5:**
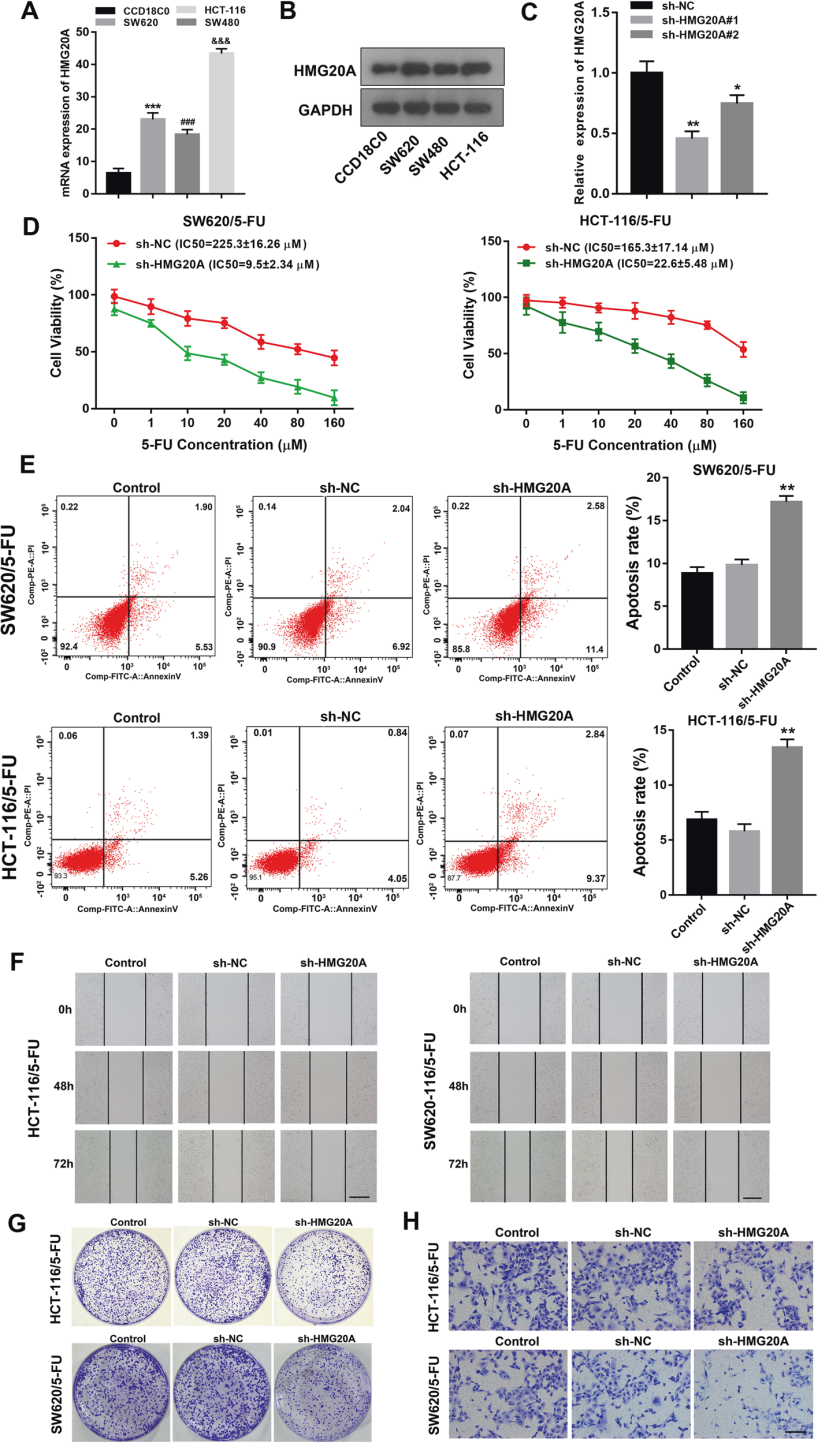
HMG20A drives 5-FU resistance and CRC proliferation, migration and invasion. The mRNA (**A**) and protein (**B**) expression of HMG20A in human colorectal epithelial cells CCD18C0 and CRC cell lines HCT-116, SW620 and SW480. **C** Expression levels to determine the efficiency of HMG20A knockdown by qRT-PCR. *P < 0.05, **P < 0.01. **D** Cell viability with indicated concentrations in HCT116/5-FU and SW620/5-FU after sh-NC or sh-HMG20A treatment by MTT assay. **E** Flow cytometry assay to measure apoptosis in HCT-116/5-FU and SW620/5-FU cells transfected with sh-NC or sh-HMG20A. **F** Wound scratch assay to measure migration in HCT-116/5-FU and SW620/5-FU cells transfected with sh-NC or sh-HMG20A. **G** Colony formation assay to measure proliferation in HCT-116/5-FU and SW620/5-FU cells transfected with sh-NC or sh-HMG20A. **H** Transwell assay to measure invasion in HCT-116/5-FU and SW620/5-FU cells transfected with sh-NC or sh-HMG20A.

Given TCGA and GEPIA database information of SFMBT1 and HMG20A in cancer, we speculated that HMG20A might be involved in colon cancer tumorigenesis and drug resistance with SFMBT1. The qRT-PCR and western blot analysis confirmed that the expression levels of *HMG20A* in CRC cells were higher than in normal epithelial colorectal cells (Fig. 5A, B, and full length uncropped original western blots in Supplementary Material). Knockdown efficiency of *HMG20A* was confirmed with qRT-PCR analysis (Fig. 5C). MTT assay indicated that the IC50 values in SW620/5-FU and HCT-116/5-FU cells after transfected with sh-NC or sh-HMG20A, the results demonstrated that silencing of HMG20A significantly affected HCT-116/5-FU and SW620/5-FU cells (Fig. 5D). Moreover, flow cytometry revealed that HMG20A knockdown up-regulated apoptosis in SW620/5-FU and HCT-116/5-FU cells (Fig. 5E). We next determined the effects of suppressing the expression of HMG20A in CRC cells. The 5-FU resistant HCT-116 and SW620 cells were transfected with sh-NC or sh-HMG20A and then migration, proliferation and invasion were assessed. Cells migrated less when HMG20A was silenced (Fig. 5F, Additional Fig. 2A). Similarly, proliferation and invasion were reduced in CRC cells after HMG20A was silenced (Fig. 5G, H, Additional Fig. 2B, C). These results indicate that the silencing of HMG20A expression suppresses CRC cell proliferation, migration and invasion.

### HMG20A and SFMBT1 interact in vitro

**Fig. 6:**
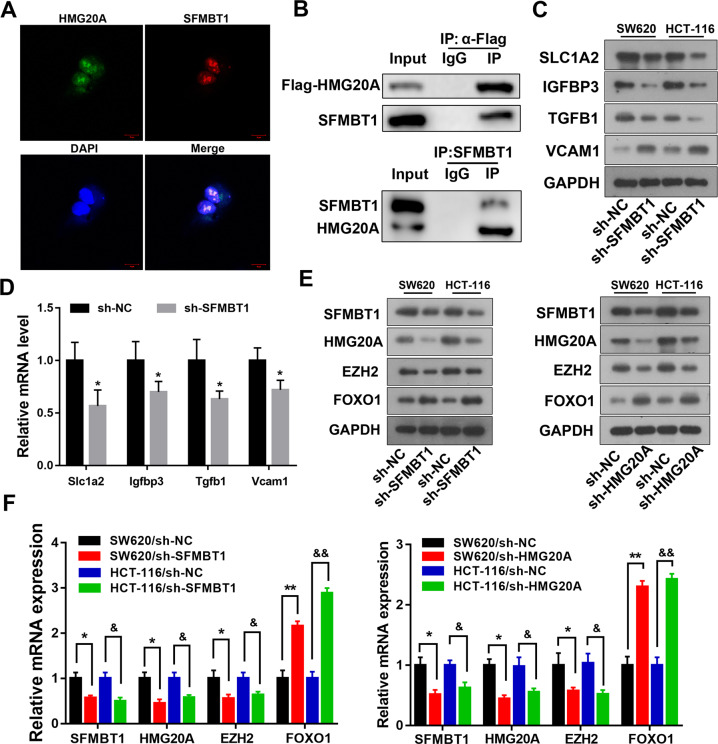
HMG20A and SFMBT1 interact in vitro. **A** After 48 h transfection, the co-localization (yellow) of HMG20A (green) and SFMBT1 (red) were analyzed using fluorescent microscopy. Cell nuclei were stained by DAPI (blue). **B** Interaction of HMG20A and SFMBT1 in cells verified by co-immunoprecipitation. **C**, **D** Western blot analysis (**C**) and qRT-PCR (**D**) detect HMG20A protein and mRNA of downstream gene and protein after SFMBT1 knockdown. **E**, **F** Western blot and qRT-PCR analysis SFMBT1, HMG20A, EZH2 and FOXO1 protein (**E**) and mRNA levels (**F**) after SFMBT1 or HMG20A knockdown in SW620 and HCT-116 cells. *P < 0.05, **P < 0.01, ^&^*P* < 0.05, ^&&^*P* < 0.01.

The GEPIA database information indicate that the SFMBT1 can interact with the HMG20A. We found that HMG20A interacted with SFMBT1 by co-IP assay and immunofluorescent staining indicates that they colocalized in the nucleus (Fig. 6A, B). To further explore whether SFMBT1 regulates HMG20A, we analyzed the transcript levels of *CTNNB1* and its target genes in SFMBT1 stably silenced HCT-116 and SW620 cells (Fig. 6C, D, and full length uncropped original western blots in Supplementary Material). The target genes of HMG20A, including *S1CLA2*, *IGFBP3*, *TGFB1* and *VCAM1* were down-regulated to different degrees when SFMBT1 knockdown. To improve our understanding of the relationship between SFMBT1 and HMG20A, we determined the expression of EZH2 and FOXO1 after SFMBT1 or HMG20A knockdown. The levels of SFMBT1, HMG20A and EZH2 were lower in cells with either SFMBT1 or HMG20A knocked down whereas levels of FOXO1 were higher (Fig. 6E, F, and full length uncropped original western blots in Supplementary Material). In all, these results verify that SFMBT1 and HMG20A could interact in the same complex to promote drug resistance in CRC.

### HMG20A in combination with SFMBT1, drive colon cancer tumorigenesis and 5-FU resistance in vivo

**Fig. 7:**
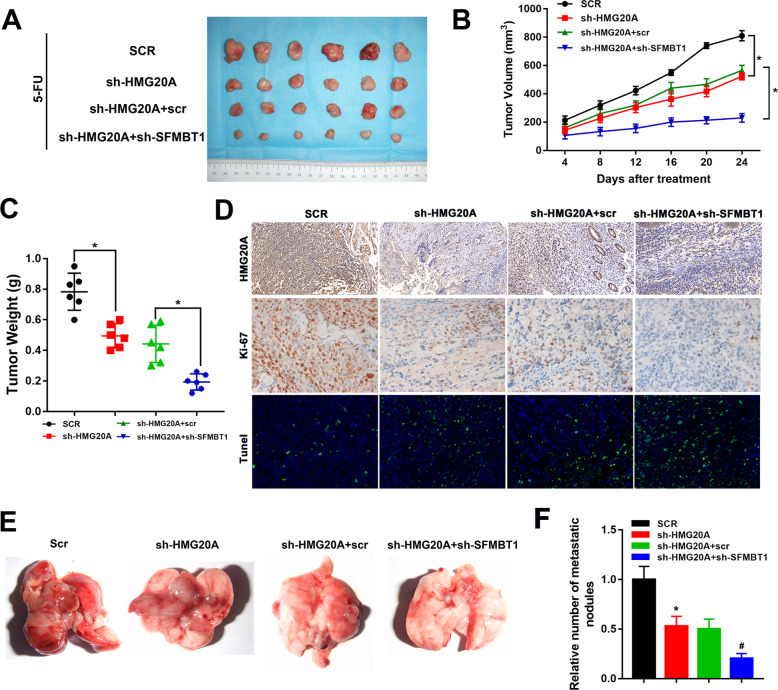
SFMBT1 in combination with HMG20A promotes the proliferation and metastasis of CRC in vivo. **A** Xenografts derived from nude mice subcutaneously injected with the indicated cells (*n* = 6). **B** Tumor volume after different treatments. **C** Tumor weight of nude mice recorded every 4 days after the initial treatment. **D** Immunohistochemistry staining of HMG20A and Ki67 and TUNEL staining of tumor tissues. Scale bars = 100 μm. **E** Representative images of lungs from each group of metastatic tissue sections. **F** The relative number of metastatic nodules were quantified. *P < 0.05, ^#^*P* < 0.05.

To investigate the function of HMG20A in combination with SFMBT1 in colon cancer in vivo, we studied xenograft tumors derived from HCT-116/5-FU cell transfected with SCR, sh-HMG20A, sh-HMG20A + scr and sh-HMG20A + sh-SFMBT1. Knockdown of HMG20A significantly reduced tumor volume and tumor growth, which were further reduced after SFMBT1 knockdown (Fig. 7A–C). Consistent with these findings, HMG20A-silenced tumors showed decreased Ki67 and increased apoptosis as indicated by IHC and TUNEL staining. When SFMBT1 is also silenced, Ki67 expression is further decreased and apoptosis is increased (Fig. 7D). Lung metastatic nodules were consistent with xenograft tumor growth (Fig. 7E, F). Consistent with in vitro experimental results, HMG20A in combination with SFMBT1 drive colon cancer tumorigenesis and 5-FU resistance in vivo.

## Discussion

Drug resistance is a major limitation to CRC survival because chemotherapeutics have harmful side effects that cannot be tolerated at high doses [25, 26]. Several studies implicate the induction of stress-related pathways as a mechanism of resistance toward the fluorouracil-based regimens used to treat CRC [27, 28]. Therefore, the modulation of genes that regulate stress-related pathways to sensitize the effects of chemotherapeutics against CRC has been the focus of recent research [29–31]. For instance, tazemetostat, an inhibitor of the histone methyltransferase EZH2, increases CRC sensitivity to 5-FU by the inhibition of EZH2. Tan et al. discovered that the inhibition of EZH2 by tazemetostat promotes the induction of PUMA by subduing reactive oxygen species and endoplasmic reticulum stress-related pathways [32].

In this study, we found that the knockdown of either SFMBT1 or HMG20A reduced the viability of CRC cells and that expression of EZH2 was also lower, whereas expression of FOXO1 was higher. In agreement with Ma et al. [14], we believe that FOXO1 is the inhibitory target gene of EZH2 and EZH2 exerts a cancer-promoting effect by inhibiting the expression of the tumor suppressor gene FOXO1. However, this interaction seems to involve the expression of either SFMBT1 or HMG20A. A co-IP assay indicated that HMG20A interacts with SFMBT1 and immunofluorescent staining suggests that SFMBT1 and HMG20A colocalize in CRC cells. Although interactions between either SFMBT1 or HMG20A and EZH2 are not well documented, there are several reports on the interactions between LSD1 and EZH2 [33–36]. For instance, an interaction between LSD1 and EZH2 is associated with the expression of interferon-stimulated genes [33]. In CRC, LSD1 and EZH2 interact to accelerate tumor progression and the suppression of either gene can modulate cell proliferation [34–36]. Carvalho et al. reported that a high expression of EZH2 was associated with disease recurrence and progression, whereas a high expression of LSD1 was associated with improved disease-free survival [37]. Lobo et al. found that the high expression of Ki67, EZH2 and SMYD3 is associated with significantly worse disease progression in prostate biopsies, whereas LSD1 was not [38]. Given that both SFMBT1 and HMG20A interact and are associated with LSD1, which in turn interacts with EZH2, we propose that EZH2 may be recruited by the HMG20A and SFMBT1 complex in CRC to down-regulate the expression of FOXO1. The down-regulation of FOXO1 promotes drug resistance and metastasis in CRC. The involvement of LSD1, perhaps through interaction with FOXO1 requires further investigation. However, these results imply that the overexpression of EZH2 and under-expression of FOXO1 are the main characteristics of CRC drug resistance and metastasis, with SFMBT1 and HMG20A identified as possible targets for therapeutic agents to increase drug resistance.

Although our study has several strengths, it is not without limitations. The dose of 5-FU used in the mouse model would not be an accurate representation of the dose delivered to patients. And the cells used to create the xenograft model were from the same source and therefore the results may not be replicated in a different genetic background, although we did assess expression levels in different CRC cell lines and similar results were obtained.

## Materials and methods

### Specimens and tissue microarray

Tissue samples of CRC patients who have not received radiotherapy or chemotherapy and corresponding normal colorectal tissue samples were obtained after all patients provided with written informed consents. Tissue microarrays of primary CRC tissues were purchased from Wuhan Google Biotechnology Co., Ltd. Our study procedures were approved by the Ethics Committee of the Ruijin Hospital Shanghai Jiao Tong University School of Medicine (No.SHDC2020CR3034).

### Animals

Twenty-four healthy male Balb/c nude mice (8 weeks old) were purchased from Shanghai Laboratory Animal Company and maintained at a 12 h day/night cycle in cages with free access to food and water. All animal experimental procedures and protocols were approved by the Laboratory Animal Ethics Committee of Ruijin Hospital Affiliated to Shanghai Jiao Tong University School of Medicine (No. GCQN2019A07). Mice were randomly and equally divided into control and treatment groups (*n* = 6), subcutaneously injected with 1 × 10^7^ cells/ml. After 4 days, and at every 4 days thereafter, tumor volume was measured. When the tumor volume reached 100 mm^3^ at 10 days, a subgroup of each treatment group received an intraperitoneal injection with 5-FU (50 mg/kg) every 4 days. After 24 days, all animals were sacrificed by cervical dislocation, and xenograft tumors were excised. Tumor size was calculated by the following: V (mm^3^) = (length × width^2^) / 2.

### Cell culture and cell transfection

The human CRC cell lines HCT-116, SW620 and SW480 and normal colorectal epithelial cells CCD18C0 were cultured in RPMI-1640 medium (Gibco BRL, Carlsbad, CA, USA) supplemented with 10% FBS and 1% penicillin-streptomycin solution in a 37 °C incubator with 5% CO_2_. 1 μM 5-FU initial concentration was induced in HCT-116 and SW620, when the cells survival rate is greater than 90%, the drug concentration (10, 20, 40 and 80 µM) is continuously increased until it can be cultured stably in 160 μM 5-FU. It took about six months to successfully construct HCT-116/5-FU and SW620/5-FU resistant cell, and the 5-FU resistant cells were cultured in 5-FU free medium for more than 2 weeks for subsequent experiments.

The following siRNA sequences were used: SFMBT1 (5′-GCAGUGGAGUCUGAAGAAUTT-3′) or HMG20A (5′-AGGCAAAUCUCAUAGGGCAA-3′). After transfection, cells were collected for subsequent experiments, and the efficiency of knockdown was determined by qRT-PCR. Cell lines were obtained from the Cell Bank of the Chinese Academy of Sciences (Shanghai, China) and authenticated by the STR method and tested for mycoplasma contamination.

### MTT assay

MTT assay was used to determine cell proliferation. Briefly, cells were seeded into 96-well plates at 5 × 10^3^ cells per well and treated with 5-FU. At the indicated time points (48 h and 72 h), 10 µl MTT solution (5 mg/ml) was added to each well and incubated at 37 °C for 4 h. Then, 150 µl DMSO was used to dissolve formazan crystals and the absorbance at 568 nm was measured.

### Clone formation

Cell proliferation was assessed by using a clone formation assay. In brief, cells were treated under specified conditions for 24 h, then cultured at a density of 3 × 10^3^ cells per dish for 14 days. After fixing the cells with ethanol for 60 min, they were stained with 5% crystal violet solution for an additional 15 min. Clones were counted under a microscope.

### Wound scratch assay

Cell migration was assessed using a wound scratch assay. CRC cells were grown to nearly 90% confluency in six-well plates. A wound was created in the cell monolayer using a 200 µL pipette tip. Sloughed cells were rinsed away with PBS. Cell migration was observed using an inverted microscope and images were taken of cells at 0, 48 and 72 h. The wound size was quantified by using Image J software.

### Transwell assay

Cell invasion assay was performed using 8 μm pore size Transwell chamber (Corning Inc., Corning, NY, USA). In brief, Matrigel (100 μl, 1 mg/ml) was added to the Transwell upper chamber. After the Matrigel had set, 200 μl of cells at a density of 4 × 10^5^ cells/ml were added to the upper chamber. After 48 h, the non-adhered cells inside the chamber were rinsed off with PBS and the invaded cells were fixed with ice ethanol for 1 h and then stained with 0.5% crystal violet for 20 min. Three fields were randomly selected and images were captured using a microscope.

### Flow cytometry assay

Apoptosis assay was measured using an Annexin V-fluorescein isothiocyanate (FITC) apoptosis detection kit (Oncogene Research Products, Boston, MA, USA) according to the manufacturer’s instructions. Briefly, 1 × 10^6^ cells were collected after incubated for 24 h and then stained with PI buffer (containing 200 lg/ml RNase and 50 lg/ml PI) for 30 min in the dark. The samples were analyzed by flow cytometry (Beckman, Brea, CA, USA).

### Hematoxylin and eosin (H&E) staining

H&E staining was used for the histological analysis of tissues. After rinsing tissues in PBS, they were fixed in 10% methanol, paraffin-embedded and sectioned (5 μm thick). The sections were deparaffinized for 10 min with xylene and a serial dilution of ethanol. They were then stained with hematoxylin for 6 min and eosin for 10 s. Images were captured using a microscope.

### Immunofluorescence

Cells were fixed in 4% paraformaldehyde for 30 min at room temperature and then blocked with 5% BSA. The blocked cells were then incubated with primary antibodies overnight at 4 °C. After washing, cells were incubated with Alexa Fluor-conjugated secondary antibodies and then stained with DAPI for 10 min. Images were captured using a Leica laser scanning confocal microscope.

### Immunoprecipitation (co-IP)

For co-IP assay, cells were lysed in RIPA buffer and then incubated with protein A/G agarose and antibody for 4 h at 4 °C. After removing debris with wash buffer, the complexes were eluted in sample buffer by placing tubes in a boiling water bath for 5 min. The eluted complexes were analyzed by western blotting.

### Real-time reverse-transcription PCR (qRT-PCR)

Total RNA was extracted by using TRIzol reagent, and cDNA was synthesized by using a PrimeScript RT Reagent Kit (TaKaRa, Kyoto, Japan). qRT-PCR was performed with an SYBR Green PCR system on a CFX96 PCR instrument (Bio-Rad, Hercules, CA, USA). *GAPDH* was used for internal normalization. The primers were listed in Supplementary Table S1.

### Western blot analysis

Total cellular protein was obtained with RIPA lysis buffer. BCA Protein Assay Kit (Beyotime, Shanghai, China) was used to measure protein concentration. Equal amounts of proteins were separated by SDS-PAGE and transferred to PVDF membranes. Membranes were blocked with 5% non-fat milk for 1 h at room temperature and then incubated with primary antibody at 4 °C overnight. Then, membranes were incubated with HRP secondary antibodies for 1 h at room temperature. Protein bands were visualized using a chemiluminescence kit (Millipore, Burlington, MA, USA) and quantitated using Image J software. Protein expression was normalized to GAPDH. SFMBT1 (PA5-22393, 1:1000), SLC1A2 (PA5-17099, 1:1000) and HMG20A (PA5-87308, 1:1000) antibodies were purchased from Invitrogen. EZH2 (#5246, 1:1000), FOXO1 (#2880, 1:1000) and IGFBP3 (#64143, 1:1000) were purchased from Cell Signaling Technology. TGFB1 (ab215715, 1:1000) and VCAM1 (ab174279, 1:2000) antibodies were purchased from Abcam.

### Statistical analysis

All experiments were replicated independently at least three times. Data were analyzed by GraphPad Prism software and expressed as the mean ± SD. One-way ANOVA test and unpaired Student’s *t* test were used to evaluate the differences among several groups or between two groups, respectively. *P* < 0.05 was considered statistically significant.

## Supplementary information

Supplemmentary Table 1

Supplement Figure 1

Supplement Figure 2

Supplementary figure legends

Original Data File

authorship change signature

## Data Availability

The datasets generated and/or analyzed during the current study are available from the corresponding author on reasonable request.
