# Supplementary material for: SFMBT1 facilitates colon cancer cell metastasis and drug resistance combined with HMG20A
Source: Cell Death Discov. 2022 May 16;8:263. doi: 10.1038/s41420-022-01057-7 (PMC9110378; doi:10.1038/s41420-022-01057-7)
Supplement: Supplementary file 1 — Supplemmentary Table 1 [file 41420_2022_1057_MOESM1_ESM.docx]

List of qRT-PCR primers

| Gene | Primer for RT-qPCR | Sequence (5’-3 ’) |
| --- | --- | --- |
| *GAPDH* | Forward | TCAAGAAGGTGGTGAAGCAGG |
|  | Reverse | TCAAAGGTGGAGGAGTGGGT |
| *SFMBT1* | Forward | CTGTCCTAACCTCTTCGGTCCACGG |
|  | Reverse | TCATCCTCATCTTCACCCCCACTTC |
| *HMG20A* | Forward | CACCCAGAGGTTCCATACAG |
|  | Reverse | CTCTGTTCATTGCCTTCTGC |
| *SLC1A2* | Forward | ACAATATGCCCAAGCAGGTAGA |
|  | Reverse | CTTTGGCTCATCGGAGCTGA |
| *TGFB1* | Forward | CTCCCGTGGCTTCTAGTGC |
|  | Reverse | GCCTTAGTTTGGACAGGATCTG |
| *IGFBP3* | Forward | GACGACGTACATTGCCTCAG |
|  | Reverse | GTCTTTTGTGCAAAATAAGGCATA |
| *VCAM1* | Forward | TTGGGAGCCTCAACGGTACT |
|  | Reverse | GCAATCGTTTTGTATTCAGGGGA |
| *EZH2* | Forward | GAGTTGGTGAATGCCCTTGG |
|  | Reverse | TGCTGTGCCCTTATCTGGAA |
| *FOXO1* | Forward | GTCCTACGCCGACCTCATC |
|  | Reverse | TGTTGCTGTCACCCTTATCCT |
