# Supplementary figures and images for: SFMBT1 facilitates colon cancer cell metastasis and drug resistance combined with HMG20A
Source: Cell Death Discov. 2022 May 16;8:263. doi: 10.1038/s41420-022-01057-7 (PMC9110378; doi:10.1038/s41420-022-01057-7)

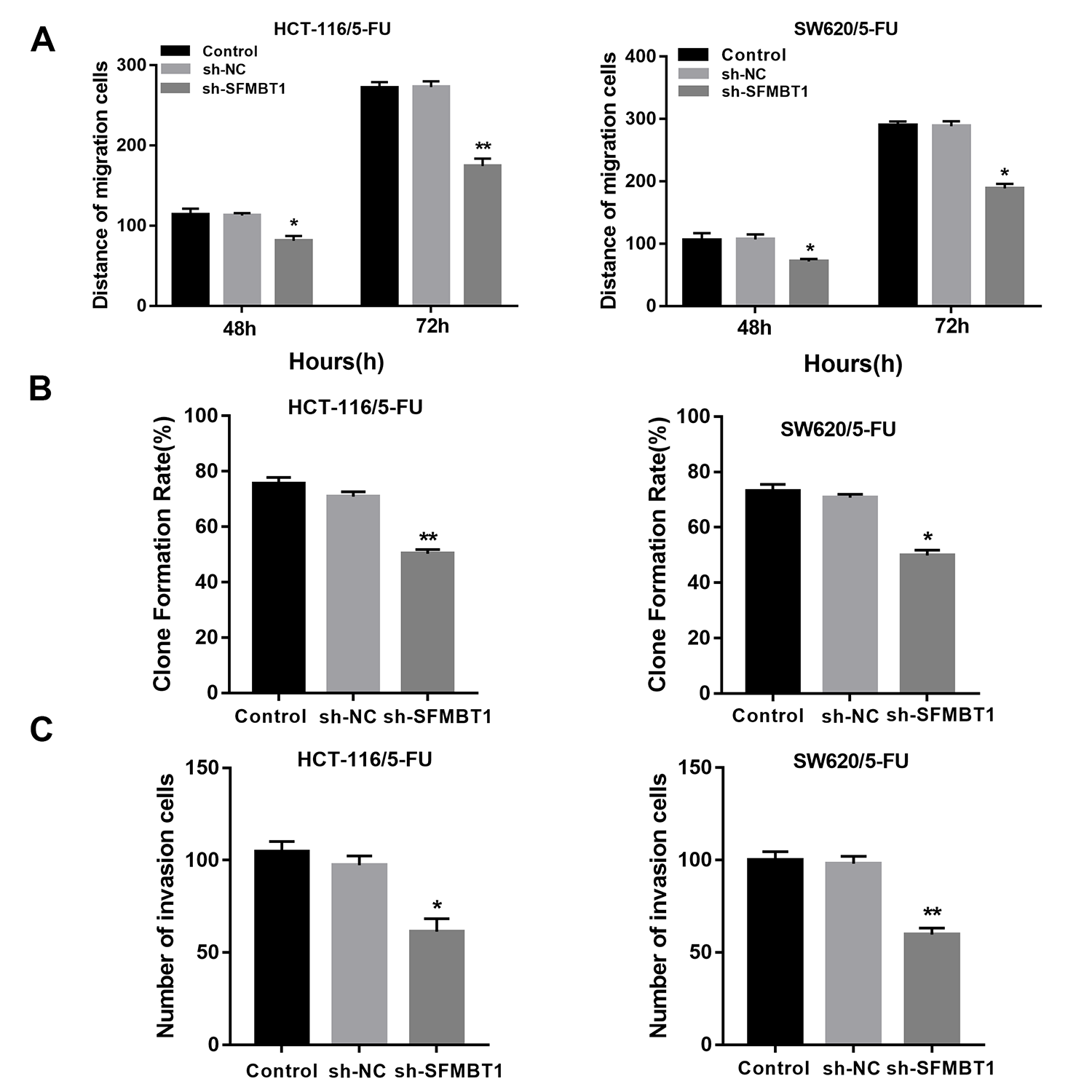

Supplement: Supplementary file 2 — Supplement Figure 1 [file 41420_2022_1057_MOESM2_ESM.tif]

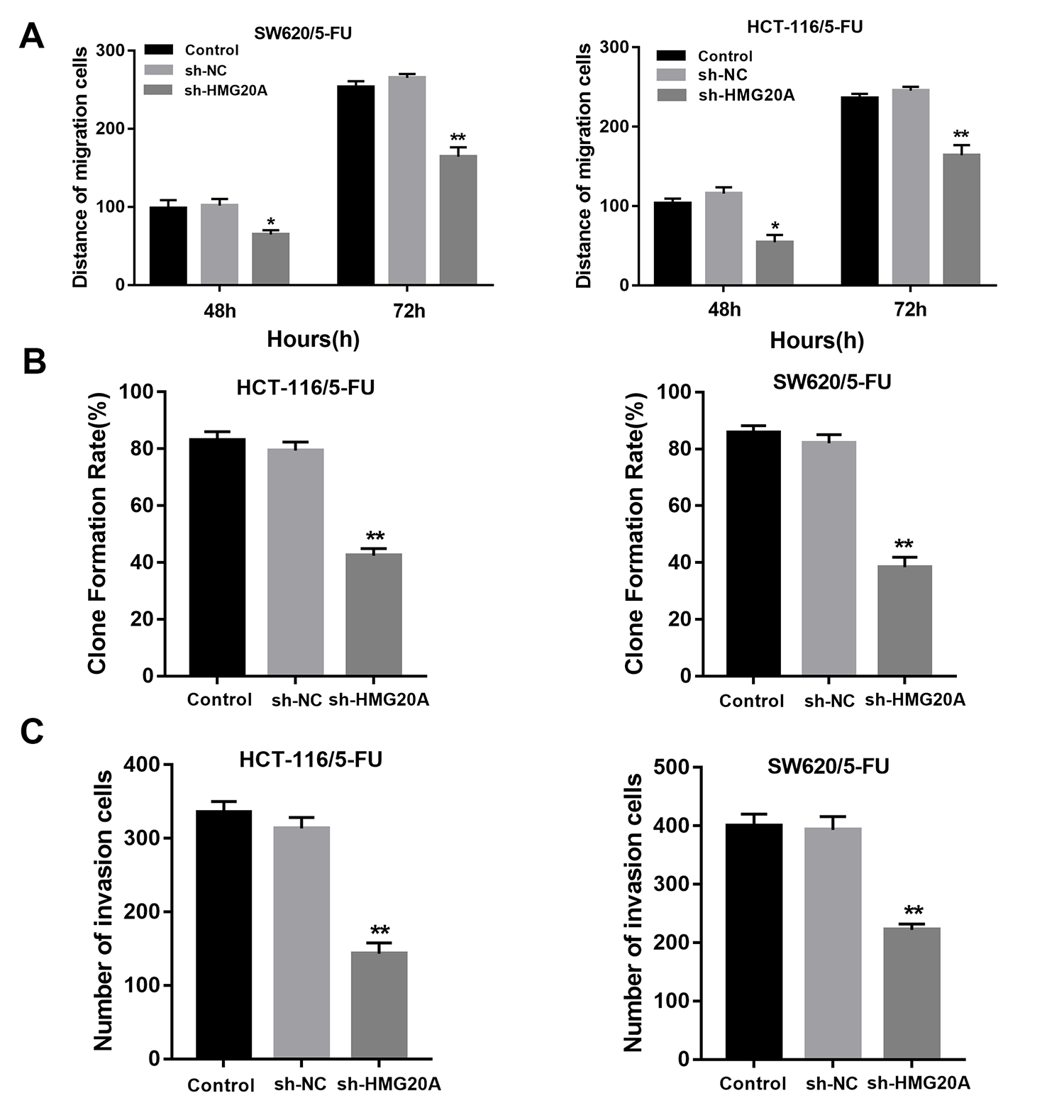

Supplement: Supplementary file 3 — Supplement Figure 2 [file 41420_2022_1057_MOESM3_ESM.tif]

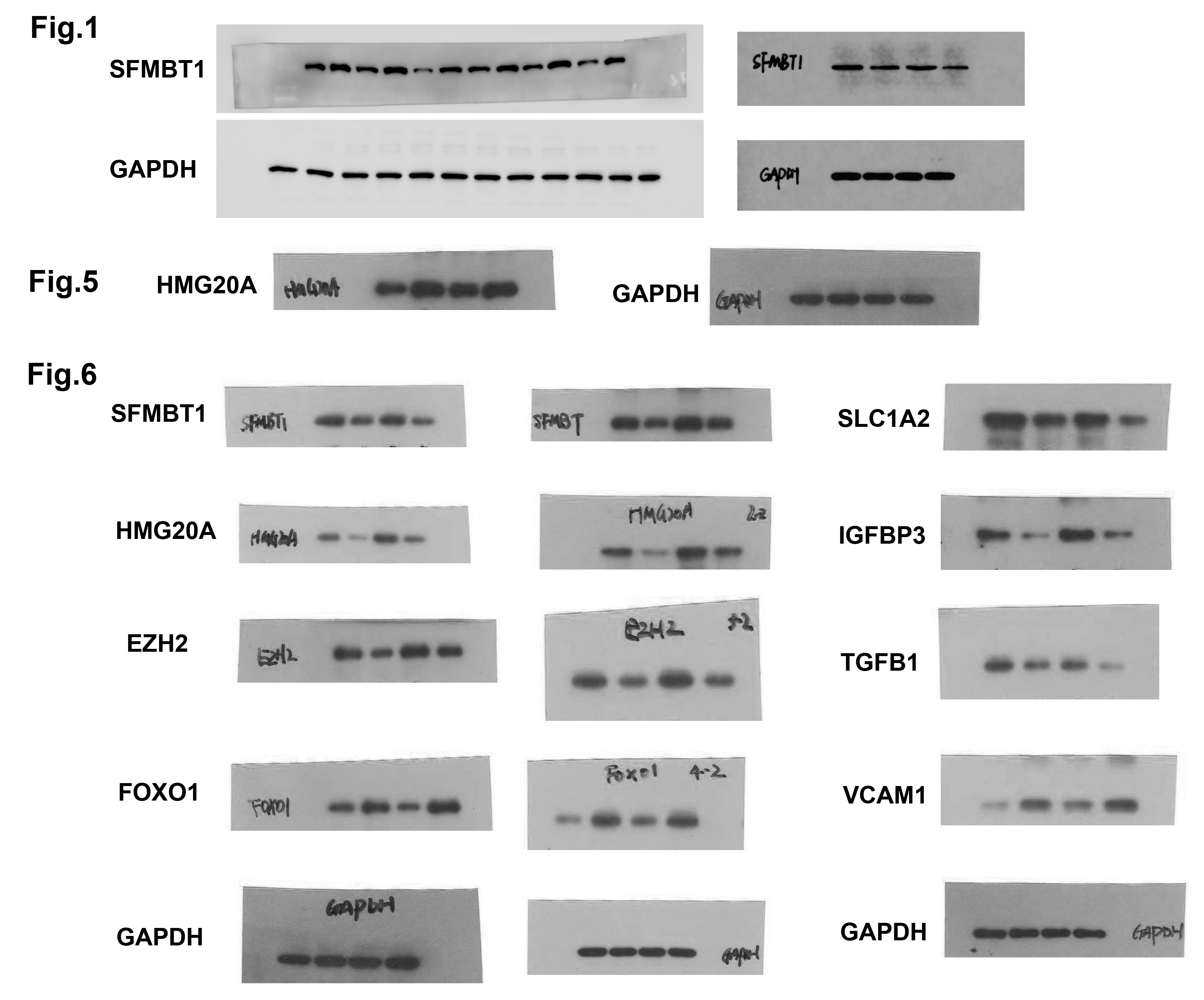

Supplement: Supplementary file 5 — Original Data File [file 41420_2022_1057_MOESM5_ESM.tif]
