# Supplementary material for: SFMBT1 facilitates colon cancer cell metastasis and drug resistance combined with HMG20A
Source: Cell Death Discov. 2022 May 16;8:263. doi: 10.1038/s41420-022-01057-7 (PMC9110378; doi:10.1038/s41420-022-01057-7)
Supplement: Supplementary file 4 — Supplementary figure legends [file 41420_2022_1057_MOESM4_ESM.docx]

**Supplementary figure legends:**

**Supplementary Figure 1.** (**A)** Wound scratch assay analysis to measure migration in HCT-116/5-FU and SW620/5-FU cells transfected with sh-NC or sh-SFMBT1. **(B)** Colony formation assay analysis to measure proliferation in HCT-116/5-FU and SW620/5-FU cells transfected with sh-NC or sh-SFMBT1. **(C)** Transwell assay analysis to measure invasion in HCT-116/5-FU and SW620/5-FU cells transfected with sh-NC or sh-SFMBT1. ^*^*P* < 0.05, ^**^*P* < 0.01.

**Supplementary Figure 2.** **(A)** Wound scratch assay analysis to measure migration in HCT-116/5-FU and SW620/5-FU cells transfected with sh-NC or sh-HMG20A. **(B)** Colony formation assay analysis to measure proliferation in HCT-116/5-FU and SW620/5-FU cells transfected with sh-NC or sh-HMG20A. **(C)** Transwell assay analysis to measure invasion in HCT-116/5-FU and SW620/5-FU cells transfected with sh-NC or sh-HMG20A. ^*^*P* < 0.05, ^**^*P* < 0.01.
