# Supplementary material for: SFMBT1 facilitates colon cancer cell metastasis and drug resistance combined with HMG20A
Source: Cell Death Discov. 2022 May 16;8:263. doi: 10.1038/s41420-022-01057-7 (PMC9110378; doi:10.1038/s41420-022-01057-7)
Supplement: Supplementary file 6 — authorship change signature [file 41420_2022_1057_MOESM6_ESM.pdf]

Dear Editor,

Thank you for your consideration for our manuscript "SFMBT1 facilitates colon cancer cell metastasis and drug resistance through HMG20A" (CDDISCOVERY-21-3278). During the revision process, **Xiao Yang** perform new experimental data such as apoptosis assay, the change of IC50 of 5-FU by sh-SFMBT1 and sh-HMG20A2 in CRC cell lines, the language revision of the paper and response the reviewers' comments. Based on **Xiao Yang**'s contributions to the paper, after consultations, and all the authors agree to replace **Zhou Zhang** with **Xiao Yang**. In the final version of the paper, the order of authors is Ruijun Pan [1,2†], Dingye Yu [1,2†], Jiajia Hu [3†], **Xiao Yang**[1,2], Chenxing Wang [1,2], Luyang Zhang [1,2], Pei Xue [1,2], Jing Sun [1,2], Xiaoping Zhang [4,5*], Wei Cai [1,2*]

Xiao Yang's institution information is below:

[1] Department of General Surgery, Ruijin Hospital, Shanghai Jiao Tong University School of Medicine

[2] Shanghai Minimally Invasive Surgery Center

All authors signed below:

Ruijun Pan Dingye Yu. Jiajia Hu

Xiao Yang Chenxing Wang

Luyang Zhang Pei Xue. Jing Sun

Wei Cai

Zhou Zhang

Xiaoping Zhang
